# Editorial: Seeing Beyond the Eye: The Brain Connection

**DOI:** 10.3389/fnins.2021.719717

**Published:** 2021-06-29

**Authors:** Christine T. O. Nguyen, Monica L. Acosta, Silvia Di Angelantonio, Thomas E. Salt

**Affiliations:** ^1^Department of Optometry and Vision Sciences, University of Melbourne, Parkville, VIC, Australia; ^2^School of Optometry and Vision Science, New Zealand National Eye Centre, Centre for Brain Research, Brain Research New Zealand - Rangahau Roro Aotearoa (BRNZ), University of Auckland, Auckland, New Zealand; ^3^Department of Physiology and Pharmacology, Sapienza University, Rome, Italy; ^4^Center for Life Nano- & Neuro- Science, Istituto Italiano di Tecnologia, Rome, Italy; ^5^Neurexpert Ltd., Newcastle upon Tyne, United Kingdom; ^6^University College London Institute of Ophthalmology, London, United Kingdom

**Keywords:** eye, brain, neurodegenenerative diseases, biomarker, retina, cortex, Alzheimer's disease, vascular disease

The eye is an anatomical extension of the brain where multiple parallels can be drawn between their neurons, vasculature and immune response. Furthermore, both organs modify similarly with disease. As such it stands to reason that multidisciplinary research that investigates both will inform upon each other, especially in the context of neurodegenerative diseases.

A rapidly emerging area of research is the use of the eye as a window to changes occurring in the brain. These range from reports of symptomatic alterations to vision, functional and structural changes in brain and retina, as well as the presence of key proteinopathy hallmarks of disease. Such findings have implications for both, the effect that neurological diseases have on vision as well as the possibility of using ocular tests as surrogate measures for detection or monitoring of systemic diseases. The eye is the only place in the body where both neurons and blood vessels can be directly visualized. These attributes have led to the common clinical practice for vascular diseases such as diabetes and hypertension, to have ocular assessment as a routine component of their management. With the advancement of *in-vivo* imaging techniques, a plethora of eye tests have been developed to reveal manifestation of systemic pathological changes in the eye. In this special issue we have curated 22 articles (15 original articles, 5 reviews, 2 perspectives) regarding the nexus between the eye and brain.

The increasing interest in this field is highlighted by manuscripts which review and provide perspectives on whether different ocular assessments may reflect neurodegenerative and vascular disease and how this may be improved in the future. This includes a systematic review on whether retinal microvasculature metrics (fractal dimensions) are modified with neurodegenerative disease and stroke (Lemmens et al.). They found a significant association with decreased retinal fractal dimension metrics and cognitive impairment/dementia. Additionally, most examined studies exhibited an association between retinal fractal dimension and acute, subacute and chronic stroke settings but more research is needed to examine the potential for prediction (Lemmens et al.).

The potential for artificial intelligence to capitalize on the data-rich capacity of eye assessments is highlighted by Li et al. and Guidoboni et al.. More specifically, a perspective article by Li et al. provides a broad overview of ocular changes that can be assessed in outpatient or “office-based” scenarios on key cognitive and psychiatric disorders. These include changes to retinal structure and microvasculature, eye-movements and pupillary responses. In particular, they highlight the role that artificial intelligence has already played in other medical fields and its potential in ocular assessment of brain disease. Guidoboni et al. expands on this concept suggesting that Artificial Intelligence coupled with mechanism-driven mathematical modeling may assist to disentangle the role that different factors play in complex diseases. Guidoboni et al. then go on to apply this approach to the fluid-filled interconnections between eye and brain as an example.

The need for more discovery research in this area is highlighted by the study by Liu et al.. In a survey of neuro-ophthalmologists they show that specialist clinicians currently find it challenging to differentiate whether visual symptoms arise from higher cortical disease or eye conditions. They highlight that better characterization of visual signs and symptoms in neurodegenerative disease will facilitate advances in the capacity for a clinician to treat a patient appropriately.

## Alzheimer's Disease

Alzheimer's disease is the most common form of dementia and yet early detection and development of new treatments continue to be a challenge. Multiple studies within this collection investigated the potential for using the eye as a window to Alzheimer's disease.

Two papers from the same group build upon interesting work in the field investigating the presence of an Alzheimer's disease hallmark, amyloid-beta, in the retina (Lee et al.; Sidiqi et al.). Lee et al. examines post-mortem retinal tissue from Alzheimer's disease patients (*n* = 15) and control eyes (*n* = 10). Focusing on the retinal ganglion cell layer they characterize the regional distribution of amyloid-beta and find higher levels in the mid-periphery and the greatest difference between Alzheimer's disease and control retinas occurring in the superior and temporal retinal quadrants. In a complementary study in rodents, they extend previous studies by other groups showing a common herbal supplement can fluorescently highlight retinal amyloid-beta and find that this measure correlates with cortical amyloid beta load, predictive of Alzheimer's disease progression (Sidiqi et al.). These works help to direct future *in-vivo* retinal amyloid-beta studies.

Animal models that recapitulate retinal and cortical amyloid-beta deposition are useful to understand the pathology associated with the presence of these protein deposits. Using Octodon degus, a rodent model that naturally accumulates amyloid-beta peptides with advancing age, Chang et al. show that synaptic remodeling, neurotransmitter changes and microglial activation occur in a time-course that parallels amyloid beta changes in the retina. Interestingly, even from a young age changes in the inner retina can be found. These inner retinal mechanistic changes are paralleled with the findings of inner retinal structure loss and dysfunction in a separate study by Lim et al.. Here *in-vivo* retinal imaging (optical coherence tomography) and electrophysiology measures indicate that the inner retina exhibits thinning of retinal ganglion cell axons and dysfunction of these neurons prior to outer retinal changes in a 5 × FAD transgenic mouse model (Lim et al.).

Also, focusing on inner retinal ganglion cells Romagnoli et al. investigate the function of a particular-type of retinal ganglion cell that is intrinsically photosensitive. This builds on previous work by their group that shows melanopsin containing retinal ganglion cells are lost or abnormal in post-mortem retinas of Alzheimer's disease donors. In the current study, they find that the chromatic pupillary response (a functional measure of melanopsin retinal ganglion cells) exhibited higher variability in mild-moderate Alzheimer's disease patients but were not significantly separated from controls (*n* = 26/group), whereas the rod mediated transient pupillary light response was altered. They hypothesize that this may reflect abnormalities in melanopsin retinal ganglion cell dendrites in the early stage of the disease before cell body manifestations.

In a proof-of-concept study that shifts ocular markers to the anterior eye, Dehghani et al. evaluate corneal dendritic cells in control, mild cognitive impairment and Alzheimer's disease patients (*n* = 5/group). Using laser scanning *in-vivo* confocal microscopy they found morphological differences including a larger dendritic cell field area and perimeter in patients with mild cognitive impairment compared to controls. These findings are consistent with an immunologically activated cell state, echoing the retinal microglial changes found in animal models by Chang et al.. Further studies evaluating corneal immunology are warranted in larger populations.

The consistent themes occurring in eye and brain are eloquently summarized by Mirzaei et al. who review the current status in the field of retina and Alzheimer's disease. Of note, multiple Alzheimer's disease pathobiological parallels have been found including the presence of pathognomic Alzheimer's proteins (plaques, amyloid-beta fibrils, protofibrils, oligomers, vascular amyloid-beta accumulation, phosphorylated Tau) as well as accompanying inflammation, pericyte loss, and neurodegeneration. Brighi et al. then extend our knowledge of the current status in the field by reviewing the literature surrounding the use of induced pluripotent stem cell-derived organoids as a model for brain and retinal tissue in a dish. Such technology may pave the way toward personalized medicines in the future.

## Vascular Disease

Dementia is thought to be not only a neurological disease but also a disease that has a vascular component. Indeed, cerebral small vessel disease can lead to multiple disorders including dementia cognitive decline and stroke. Neuroimaging features of cerebral small vessel disease include recent small subcortical infarcts and white matter hyper-intensities. Cao et al. found in 40 recent small subcortical infarct patients vs. 46 controls that optical coherence tomography angiography was reduced in the macula area (both superficial and deep retinal capillary plexus) and around the optic nerve head (radial peripapillary capillary bed) which correlated with peripapillary retinal nerve fiber layer thickness. In another study, Peng et al. assessed optical coherence tomography angiography on 74 participants with white matter hyper-intensities who were stroke and dementia free at the time of assessment. They found that microvascular density around the optic nerve (radial peripapillary capillaries) and the deep capillary plexus at the macula were associated with disease severity and cognitive function. These two studies lay a foundation for use of optical coherence tomography angiography to evaluate vasculature in patients with cerebral small vessel disease pending longitudinal studies with larger sample sizes. Interestingly, in a pilot functional MRI study that longitudinally examined patients recovering from stroke (*n* = 15 chronic stroke patients, *n* = 12 controls) Wu et al. found that the sensorimotor network connectivity played a facilitatory role following stroke rehabilitation. They suggested this could be used as a rehabilitation biomarker for future development of interventions and treatments. These studies are in alignment with the aforementioned systematic review which indicated for an association between retinal microvasculature integrity (fractal dimensions) and stroke (Lemmens et al.).

Provost et al. extend previous assessment of retinal vasculature in adults to children. They quantify retinal microvasculature indices, including vessel diameter, fractal dimension, lacunarity and tortuosity, from fundus photographs of 190 children. They found that measures reflecting a denser microvascular network, high fractal dimension and low lacunarity, were associated with poorer behavioral and attention outcomes in children assessed via tests from the Neurobehavioral Evaluation System battery. This association of higher retinal fractal dimension has also been shown in adult patients with schizophrenia and bipolar disorder. In contrast, the aforementioned studies indicate for a sparser retinal density with cerebral small vessel disease (Cao et al.; Lemmens et al.; Peng et al.) associated with a cognitive decline. Whether these differences reflect distinct mechanisms between these diseases or a different time-course of changes (initial upregulation with subsequent loss for instance) requires further investigation.

Looking at vascular disease from a flipped perspective (eye disease manifesting in the brain) Huang et al. evaluate diabetic retinopathy patients and conduct dynamic cerebral activity imaging (dynamic amplitude of low frequency fluctuation) as a more sensitive measure than conventional static cerebral activity measures. Here they find that compared with healthy controls (*n* = 38) diabetic retinopathy patients (*n* = 34) exhibited significantly increased variability in their dynamic cerebral activity imaging measure in the visual cortex, cerebellum and parahippocampal gyrus.

## Other Neurodegenerative Disease

Following Alzheimer's disease, Parkinson's disease is the second most common neurological disorder.

Indrieri et al. provide a comprehensive review of visual changes and parallel molecular mechanisms in Parkinson's disease. Altered visual measures include reduced visual acuity, contrast sensitivity, color vision and more complex visual processing tasks. The retinal manifestations of dopamine, alpha-synuclein and mitochondrial dysfunction are also reviewed. Together these have been shown to manifest as changes in objective assessments such as optical coherence tomography and electroretinography, but the authors suggest that coupling these measures with future imaging of retinal alpha-synuclein will improve specificity.

Shen et al. characterize the retinal phenotype of neuromyelitis optica spectrum disorder, an autoimmune inflammatory disease of the central nervous system. More specifically, the BDNF Val66Met genetic polymorphism was evaluated in this study given its known links to neuronal health. In 17 neuromyelitis optica spectrum disorder patients they found the BDNF Val66Met was associated with more severe nerve fiber layer damage using optical coherence tomography imaging in optic neuritis eyes. Met carriers had thinner OCT layers that corresponded to ganglion cells and delayed multifocal VEP in optic neuritis eyes. In contrast no associations were found in non-optic neuritis eyes suggesting that the gene may be associated with optic neuritis and local damage to the optic nerve in these patients.

Studies in animal models facilitate insight into pathobiological changes that are occurring in disease. Tyro3 is a tyrosine kinase receptor that is expressed in the brain and more recently has also been shown in the retina. Blades et al. use a Tyro3 knock out mouse model to investigate the role of Tyro3 on structure and function of the retina. They find that Tyro3 deficiency causes a reduction in function across the retina, including from photoreceptor, bipolar, retinal ganglion cells. Of note, the decrease in retinal ganglion cell function was paralleled by *in-vivo* thinning of ganglion cell axons (assessed via optical coherence tomography) and *ex-vivo* assays of retinal ganglion cell health (a reduced number of retinal ganglion cells and fewer dendrites in the ON-retinal ganglion cell layer). Further studies are warranted to examine whether Tyro3 pathways could inform new therapeutic approaches for ganglion cell diseases.

Duchenne muscular dystrophy is a muscular disease, but patients also suffer from cognitive impairment and visual defects. It is caused by problems with the cytoskeletal protein dystrophin, Dp427 which are expressed on selected neurons including those in the retina. By employing biochemical and immunobiological approached this study (Persiconi et al.) shows for the first time that Dp427 is involved in the retinal neurodevelopment process, as reported previously for other central nervous system neurons. Interestingly, in the retina neural alteration during development may be compensated during growth and underlie adult physiological dysfunctions, a role distinct to the retina. This work in conjunction with further functional characterization will inform our understanding of vision changes in Duchenne muscular dystrophy.

Agadagba et al. investigate the effect of transcorneal electrical stimulation on a retinitis degeneration mouse model. They find that this treatment induced both local (electrocorticogram recordings from primary visual cortex) and global (electrocorticogram recordings from prefrontal cortex) cortical excitation in a manner that modifies with stimulation frequency. These findings may have implications for retinal prostheses to treat retinal degeneration in the future. Together these preclinical studies (Agadagba et al.; Blades et al.; Persiconi et al.) form a foundation for our understanding of the potential for novel interventions to treat retinal or cortical disease.

In summary, this collection of articles brings together some notable recent advances in the field. It shows that brain changes are reflected in the eye and vice versa. The challenge for the field moving forward may lie in determining whether there are specific changes that occur in the eye for different diseases. Together these articles indicate that neurological diseases may affect the anatomy and function of the eye. Studies like these form the basis for continuing research aiming at understanding the plethora of eye and brain connections.

## Author Contributions

CN, MA, SD, and TS reviewed the submissions, wrote the editorial, and approved it for publication. All authors contributed to the article and approved the submitted version.

## Conflict of Interest

CN is a Chief Investigator on an Australian Research Council Linkage grant LP160100126 with AstraZeneca Neuroscience and Biogen Inc. TS is the Director and Chief Scientific Officer of Neurexpert Ltd. The remaining authors declare that the research was conducted in the absence of any commercial or financial relationships that could be construed as a potential conflict of interest.

